# Association of Checklist Use in Endotracheal Intubation With Clinically Important Outcomes: A Systematic Review and Meta-analysis

**DOI:** 10.1001/jamanetworkopen.2020.9278

**Published:** 2020-07-02

**Authors:** Joseph S. Turner, Antonino W. Bucca, Steven L. Propst, Timothy J. Ellender, Elisa J. Sarmiento, Laura M. Menard, Benton R. Hunter

**Affiliations:** 1 Department of Emergency Medicine, Indiana University School of Medicine, Indianapolis; 2 Now with Department of Emergency Medicine, CoxHealth, Springfield, Missouri; 3 Ruth Lilly Medical Library, Indiana University School of Medicine, Indianapolis

## Abstract

**Importance:**

Endotracheal intubation of critically ill patients is a high-risk procedure. Checklists have been advocated to improve outcomes.

**Objective:**

To assess whether the available evidence supports an association of use of airway checklists with improved clinical outcomes in patients undergoing endotracheal intubation.

**Data Sources:**

For this systematic review and meta-analysis, PubMed (OVID), Embase, Cochrane, CINAHL, and SCOPUS were searched without limitations using the Medical Subject Heading terms and keywords *airway*; *management*; *airway management*; *intubation, intratracheal*; *checklist*; and *quality improvement* to identify studies published between January 1, 1960, and June 1, 2019. A supplementary search of the gray literature was performed, including conference abstracts and clinical trial registries.

**Study Selection:**

Full-text reviews were performed to determine final eligibility for inclusion. Included studies were randomized clinical trials or observational human studies that compared checklist use with any comparator for endotracheal intubation and assessed 1 of the predefined outcomes.

**Data Extraction and Synthesis:**

Data extraction and quality assessment were performed using the Newcastle-Ottawa Scale for observational studies and Cochrane risk of bias tool for randomized clinical trials. Study results were meta-analyzed using a random-effects model. Reporting of this study follows the Preferred Reporting Items for Systematic Reviews and Meta-analyses (PRISMA) reporting guideline.

**Main Outcomes and Measures:**

The primary outcome was mortality. Secondary outcomes included first-pass success and known complications of endotracheal intubation, including esophageal intubation, hypoxia, hypotension, and cardiac arrest.

**Results:**

The search identified 1649 unique citations of which 11 (3261 patients) met the inclusion criteria. One randomized clinical trial and 3 observational studies had a low risk of bias. Checklist use was not associated with decreased mortality (5 studies [2095 patients]; relative risk, 0.97; 95% CI, 0.80-1.18; *I*^2^ = 0%). Checklist use was associated with a decrease in hypoxic events (8 studies [3010 patients]; relative risk, 0.75; 95% CI, 0.59-0.95; *I*^2^ = 33%) but no other secondary outcomes. Studies with a low risk of bias did not demonstrate decreased hypoxia associated with checklist use.

**Conclusions and Relevance:**

The findings suggest that use of airway checklists is not associated with improved clinical outcomes during and after endotracheal intubation, which may affect practitioners’ decision to use checklists in this setting.

## Key Points

**Question:**

Is the use of airway checklists associated with improved outcomes in patients undergoing endotracheal intubation?

**Findings:**

This systematic review and meta-analysis of 11 studies with 3261 patients undergoing endotracheal intubation did not find a difference in mortality or most secondary outcomes associated with checklist use.

**Meaning:**

The findings suggest that the use of airway checklists during endotracheal intubation is not associated with improved outcomes.

## Teaser

This systematic review and meta-analysis assesses whether the use of airway checklists is associated with improved outcomes in patients undergoing endotracheal intubation.

## Introduction

Endotracheal intubation (ETI) is a frequently used life-saving procedure. In the US annually, 15 million operating room intubations and 650 000 hospital intubations outside the operating room are performed, including 346 000 emergency department (ED) intubations.^1,2^ Despite its frequency, ETI is a high-risk procedure, with significant rates of respiratory complications, hemodynamic instability, and cardiac arrest.^3,4,5,6,7^ Interventions to improve the safety and success of ETI could thus have a substantial effect on public health. Checklists are a form of cognitive strategy intended to force operators to ensure appropriate preparation before a procedure. Checklists have been associated with improved outcomes in multiple aspects of health care^8,9,10,11,12,13^ and have been endorsed as a means to reduce complications during ETI.^6,14,15,16^

The theoretical benefits of checklist use must be balanced with potential risks. Checklist adoption often faces numerous barriers and may require a substantial investment of time and resources.^17,18^ Checklist fatigue may occur with checklist endorsement for multiple different procedures.^19^ Furthermore, checklists are not universally correlated with improved outcomes^18,20^ and, in some cases, have even been associated with harm.^21^ This study evaluated the association between checklist use and clinical outcomes after ETI.

## Methods

This systematic review and meta-analysis followed the Preferred Reporting Items for Systematic Reviews and Meta-analyses (PRISMA) reporting guideline. The protocol has been published on PROSPERO (CRD42019140071).

### Data Sources and Search Strategy

PubMed (OVID), Embase, Cochrane, CINAHL, and SCOPUS were searched without limitations to identify studies published between January 1, 1960, and June 1, 2019. The following Medical Subject Heading terms and keywords were identified collaboratively between 2 of us (J.S.T., L.M.M.) (the eAppendix in the Supplement gives the search details): *airway*; *management*; *airway management*; *intubation, intratracheal*; *checklist*; and *quality improvement*. In addition, a supplementary search of the gray literature was performed, including conference abstracts and clinical trial registries, but only peer-reviewed publications were eligible for inclusion. Bibliographies of included studies and relevant reviews were hand searched, and experts in the field were queried to identify additional studies.

### Study Eligibility Criteria and Study Selection

Included studies met the following criteria: (1) evaluated an airway checklist regardless of checklist content in patients being intubated in any setting (protocols or procedures that did not use a checklist were not included), (2) included a comparator group without checklist use, and (3) assessed at least 1 of the predefined outcomes. Simulation studies or studies with no comparator group or no assessment of the outcomes of interest were excluded.

After the removal of duplicates, all titles and abstracts identified by the search were screened independently by 2 of us (J.S.T., A.W.B.). Full text was obtained for all articles deemed to be possibly relevant by either screener. Full-text reviews were performed independently by 2 of us (S.L.P., B.R.H.) to determine final eligibility for inclusion in the review. Disagreements about inclusion were resolved through discussion. If additional information was needed to determine eligibility, we attempted to contact the corresponding authors for individual studies.

### Quality Appraisals

Risk of bias of the included studies was assessed using 2 different quality assessment tools. Randomized clinical trials were assessed by the Cochrane risk of bias tool.^22^ In brief, each study was assigned a high, low, or unclear risk of bias in each of 7 domains: random sequence generation, allocation concealment, blinding of participants and caregivers, blinding of outcome assessors, attrition bias, incomplete outcome bias, and other bias. Observational studies were assessed using the Newcastle-Ottawa Scale.^23^ This scale assigns up to 4 points for low risk of bias in the domain of selection of patients and comparators, 2 possible points for comparability, and 3 possible points for low risk of bias in determination of exposure. Studies are thus awarded between 0 and 9 points, with higher scores indicating lower risk of bias. Each study underwent quality assessment by 2 of us (S.L.P., B.R.H.) independently, with disagreements resolved through discussion.

### Data Extraction

Two of us (J.S.T., A.W.B.) independently extracted data from each study. Data abstracted included year of publication, country, clinical setting, study design, inclusion and exclusion criteria, components of used checklists, number of patients, comparator interventions, and primary outcomes. For each study, the number of patients with and without each of our predefined outcomes was calculated for patients for whom a checklist was used and for the control group. Any discrepancies were resolved through discussion. In cases of missing data or need for clarification, we contacted corresponding authors of the original studies.

### Outcomes

The primary outcome was mortality. We chose mortality as a primary outcome because it is the most patient-important outcome, and ETI is often performed on patients with significant risk of death. Mortality was recorded according to how it was reported in individual studies. If multiple measures of mortality were given, hospital mortality was used preferentially. Other outcomes of interest included rates of hypoxia, rates of hypotension, first-pass intubation success, time to successful intubation, peri-intubation arrest, esophageal intubation, and hospital length of stay. First-attempt intubation success was defined as successful ETI before removing the laryngoscope from the patient’s mouth. Peri-intubation arrest was defined as any loss of pulses that required cardiopulmonary resuscitation or defibrillation within 60 minutes after ETI. We allowed hypoxia and hypotension to be defined as described in individual studies because more granular data were not available. We performed preplanned sensitivity analyses of all included outcomes, including only studies with a low risk of bias. Subgroup analyses were performed for pediatric vs adult studies and ED vs intensive care unit (ICU) studies.

### Statistical Analysis

Study results were meta-analyzed using a random-effects model to generate the summary relative risk (RRs) with corresponding 95% CIs. Heterogeneity (*I*^2^ and *P* values) were also reported. A 2-sided *P* < .05 was considered to be statistically significant. Statistical analyses were performed using the metan module of StataMP, version 16 (StataCorp LLC).

## Results

**Figure 1.**
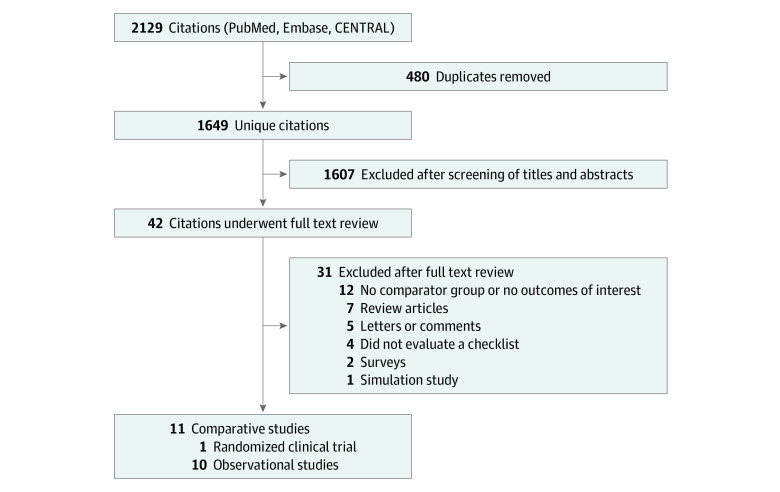
Study Identification

Figure 1 outlines the flow of study identification. The initial database search returned 1649 unique citations. After screening of titles and abstracts, 1607 citations were excluded, and 42 articles underwent full-text review, with 11 meeting inclusion criteria.^1,24,25,26,27,28,29,30,31,32,33^ Reasons for exclusion are listed in Figure 1. We requested clarification about inclusion criteria from the authors of 2 studies.^25,34^ Both studies described the use of an intubating protocol without specifying whether a checklist was used. We were able to confirm that a checklist was used in the study by Corl et al,^25^ but we were unable to confirm the use of a checklist for the other study,^34^ which was thus excluded.

### Characteristics of Studies and Patients

Across the 11 included studies,^1,24,25,26,27,28,29,30,31,32,33^ there were a total of 3261 ETIs performed in 13 institutions in 6 countries. Table 1 gives the characteristics of the individual studies. Seven studies^1,24,26,29,30,31,32^ were conducted in the ED, 3 studies^25,27,28^ in the ICU, and 1 study^33^ in both the operating room and ICU. Only 1 (ICU-based) study^28^ was a randomized clinical trial. Eight studies^24,25,26,27,29,30,32,33^ used a before-and-after observational design, with 6 studies^25,26,29,30,32,33^ being prospective and 2 studies^24,27^ being retrospective. The remaining 2 studies^1,31^ were prospective case series. None of the observational studies attempted to correct for baseline differences between groups. Five studies^25,26,27,29,30^ included significant cointerventions (such as health care professional education, equipment changes, health care professional team modeling, and medication changes) in addition to a checklist. Preintubation checklist details were reported in 8 studies^24,25,26,27,28,29,32,33^ and were heterogeneous. Most checklists included assessment of preoxygenation and medication, but other checklist components were inconsistent (eTable in the Supplement).

**Table 1.** Study Characteristics

Source	Study type	Country	Setting	Patients, No.	Outcomes assessed
Conroy et al,^24^ 2014	Retrospective before and after	US	ED	187	Mortality and FPS
Corl et al,^25^ 2018	Prospective before and after	US	ICU	275	Mortality, FPS, hypoxia, hypotension, cardiac arrest, EI, and hospital LOS
Fogg et al,^26^ 2016	Prospective before and after	Australia	ED	655	FPS, hypoxia, hypotension, cardiac arrest, and EI
Hatch et al,^27^ 2016	Retrospective before and after	US	Neonatal ICU	509	Mortality, hypoxia, TTI, hypotension, cardiac arrest, and EI
Janz et al,^28^ 2018	Randomized clinical trial	US	ICU	262	Mortality, FPS, hypoxia, TTI, hypotension, cardiac arrest, and EI
Kerrey et al,^29^ 2015	Prospective before and after	US	Pediatric ED	189	Hypoxia
Lewis et al,^1^ 2018	Prospective case series	South Africa	ED and prehospital	41	FPS
Long et al,^30^ 2017	Prospective before and after	Australia	Pediatric ED	117	FPS, hypoxia, and hypotension
Powell et al,^31^ 2018	Prospective case series	New Zealand	ED	23	FPS
Smith et al,^32^ 2015	Prospective before and after	US	ED	141	FPS, hypoxia, hypotension, TTI, cardiac arrest, and EI
Szucs et al,^33^ 2019	Prospective before and after	Hungary	ICU and OR	862	Mortality, FPS, hypoxia, hypotension, and cardiac arrest

Abbreviations: ED, emergency department; EI, esophageal intubation; FPS, first-pass success; ICU, intensive care unit; LOS, length of stay; OR, operating room; TTI, time to intubation.

Definitions of hypoxia and hypotension varied among the studies. Definitions of hypoxia ranged from less than 60% to less than 93%, with a median of 90%. Two studies^26,27^ defined hypotension as a decrease in blood pressure that required intervention with fluid bolus or vasopressor. Other studies^25,28,30,32,33^ used definitions that included a systolic blood pressure from 70 to 90 mm Hg.

The only randomized clinical trial^28^ had a high risk of bias for blinding because neither practitioners nor outcome assessors were blinded to treatment group. Allocation concealment had an unclear risk of bias. The study^28^ had a low risk for random sequence generation, attrition bias, incomplete outcomes, and other bias. This study^28^ was included in the low risk of bias sensitivity analyses.

Table 2 outlines the risk of bias assessments for each domain of the Newcastle-Ottawa Scale across all 10 included observational studies.^1,24,25,26,27,29,30,31,32,33^ Of 9 possible stars awarded in the Newcastle-Ottawa Scale, scores ranged from 4 to 8 stars.

**Table 2.** Newcastle-Ottawa Scale Scores for Observational Studies

Source	Selection			Comparability			Outcome		
	Exposed cohort representative	Selection of unexposed	Exposure ascertainment blind or objective?	Outcome not present pre-exposure?	Controlled for other interventions/important confounders?	Similar baseline demographics or other confounders?	Outcome assessment blind or objective?	Follow-up long enough?	Lost to follow-up
Conroy et al,^24^ 2014	Low risk of bias	High risk of bias	Low risk of bias	Low risk of bias	Low risk of bias	Low risk of bias	Low risk of bias	Low risk of bias	Low risk of bias
Corl et al,^25^ 2018	Low risk of bias	High risk of bias	Low risk of bias	Low risk of bias	High risk of bias	Low risk of bias	High risk of bias	Low risk of bias	Low risk of bias
Fogg et al,^26^ 2016	Low risk of bias	High risk of bias	Low risk of bias	High risk of bias	High risk of bias	High risk of bias	High risk of bias	Low risk of bias	Low risk of bias
Hatch et al,^27^ 2016	Low risk of bias	High risk of bias	Low risk of bias	High risk of bias	High risk of bias	Low risk of bias	Low risk of bias	Low risk of bias	No
Kerrey et al,^29^ 2015	Low risk of bias	High risk of bias	Low risk of bias	High risk of bias	High risk of bias	High risk of bias	Low risk of bias	Low risk of bias	Low risk of bias
Lewis et al,^1^ 2018	Low risk of bias	High risk of bias	Low risk of bias	Low risk of bias	High risk of bias	High risk of bias	High risk of bias	Low risk of bias	Low risk of bias
Long et al,^30^ 2017	Low risk of bias	High risk of bias	Low risk of bias	High risk of bias	High risk of bias	High risk of bias	High risk of bias	Low risk of bias	Low risk of bias
Powell et al,^31^ 2018	Low risk of bias	Low risk of bias	Low risk of bias	Low risk of bias	High risk of bias	High risk of bias	High risk of bias	Low risk of bias	Low risk of bias
Smith et al,^32^ 2015	Low risk of bias	High risk of bias	Low risk of bias	High risk of bias	Low risk of bias	Low risk of bias	Low risk of bias	Low risk of bias	Low risk of bias
Szucs et al,^33^ 2019	Low risk of bias	High risk of bias	Low risk of bias	Low risk of bias	Low risk of bias	Low risk of bias	Low risk of bias	Low risk of bias	Low risk of bias

We defined overall low risk of bias for observational studies^24,32,33^ as at least 7 of 9 possible stars. Three studies^24,32,33^ met these criteria. The remaining observational studies,^1,25,26,27,29,30,31^ with 4 to 6 stars, were deemed to have high to moderate risk of bias.

### Main Results

**Figure 2.**
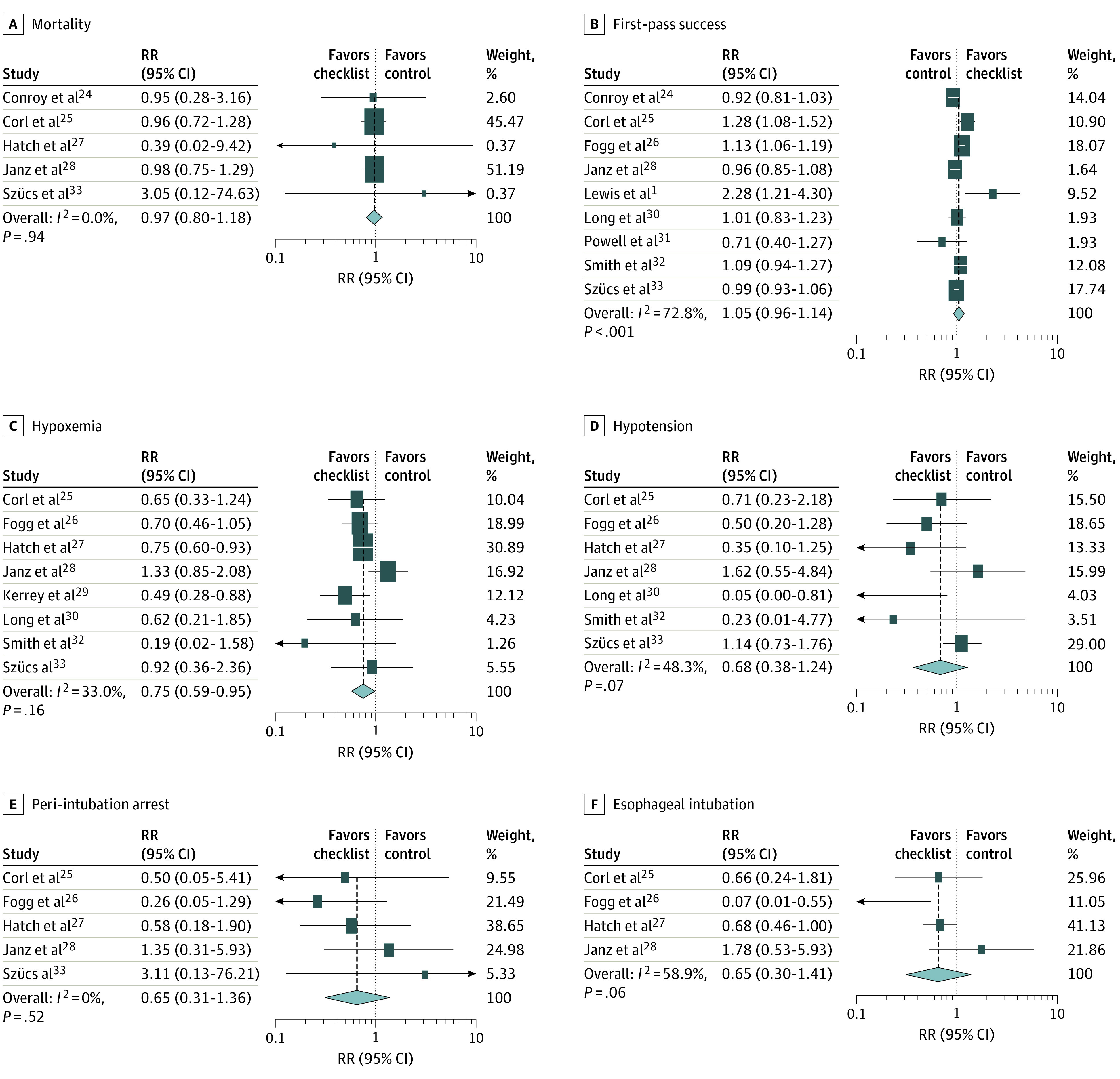
Summary Estimates of Relative Risks (RRs) for Binary Outcomes Squares indicate RR estimates, with horizonal lines representing 95% CIs. Diamonds represent pooled estimates, with points indicating 95% CIs. Shaded boxes represent the contribution weight of each study to the meta-analysis. Vertical dashed lines represent the relationship of the 95% CIs around each individual study result with the pooled mean. Weights are from random-effects analysis. A, E, and F, The study by Smith et al^32^ was not included in the analysis.

Forest plots with summary estimates of RRs and 95% CIs for binary outcomes are displayed in Figure 2. For the primary outcome, mortality was reported in 5 studies^24,25,27,28,33^ with 2095 patients. The pooled mortality rate was 11.3%. No association was found between mortality and preintubation checklist use (pooled RR, 0.97; 95% CI, 0.80-1.18), with low heterogeneity (*I*^2^ = 0%).

Among secondary outcomes, checklist use was not associated with a statistically significant difference in the rate of most adverse events, including esophageal intubation (4 studies^25,26,27,28^ [1701 patients]; RR, 0.65; 95% CI, 0.30-1.41; *I*^2^ = 58.9%), hypotension (7 studies^25,26,27,28,30,32,33^ [2821 patients]; RR, 0.68; 95% CI, 0.38-1.24; *I*^2^ = 48.3%), or peri-intubation cardiac arrest (5 studies^25,26,27,28,32^ [2563 patients]; RR, 0.65; 95% CI, 0.31-1.36; *I*^2^ = 0%). However, checklist use was associated with a decrease in hypoxic events (8 studies^25,26,27,28,29,30,32,33^ [3010 patients]; RR, 0.75; 95% CI, 0.59-0.95; *I*^2^ = 33%). This association was more pronounced in studies^26,29,30,32,33^ with cutoffs for hypoxia of 90% to 93% (eFigure 1 in the Supplement). Checklist use was also not associated with increased first-pass intubation success (9 studies^1,24,25,26,28,30,31,32,33^ [2563 patients]; RR, 1.05 with checklist; 95% CI, 0.96-1.14; *I*^2^ = 73%) (Figure 2). Time to successful intubation results were not pooled because definitions differed among the studies. Smith et al^32^ reported decreased time from paralysis to intubation associated with checklist use (82 vs 94 seconds, *P* = .02). Janz et al^28^ reported no difference in time from induction to intubation (120 seconds with checklist and 118 seconds without). Lastly, Hatch et al^27^ reported an increase in time from decision to intubate to successful intubation associated with checklist use (33 vs 27 minutes, *P* = .01). Hospital length of stay was only reported in 1 study^25^ and was not different between groups (11 days for checklist group and 12 days for control group, *P* = .55).

**Figure 3.**
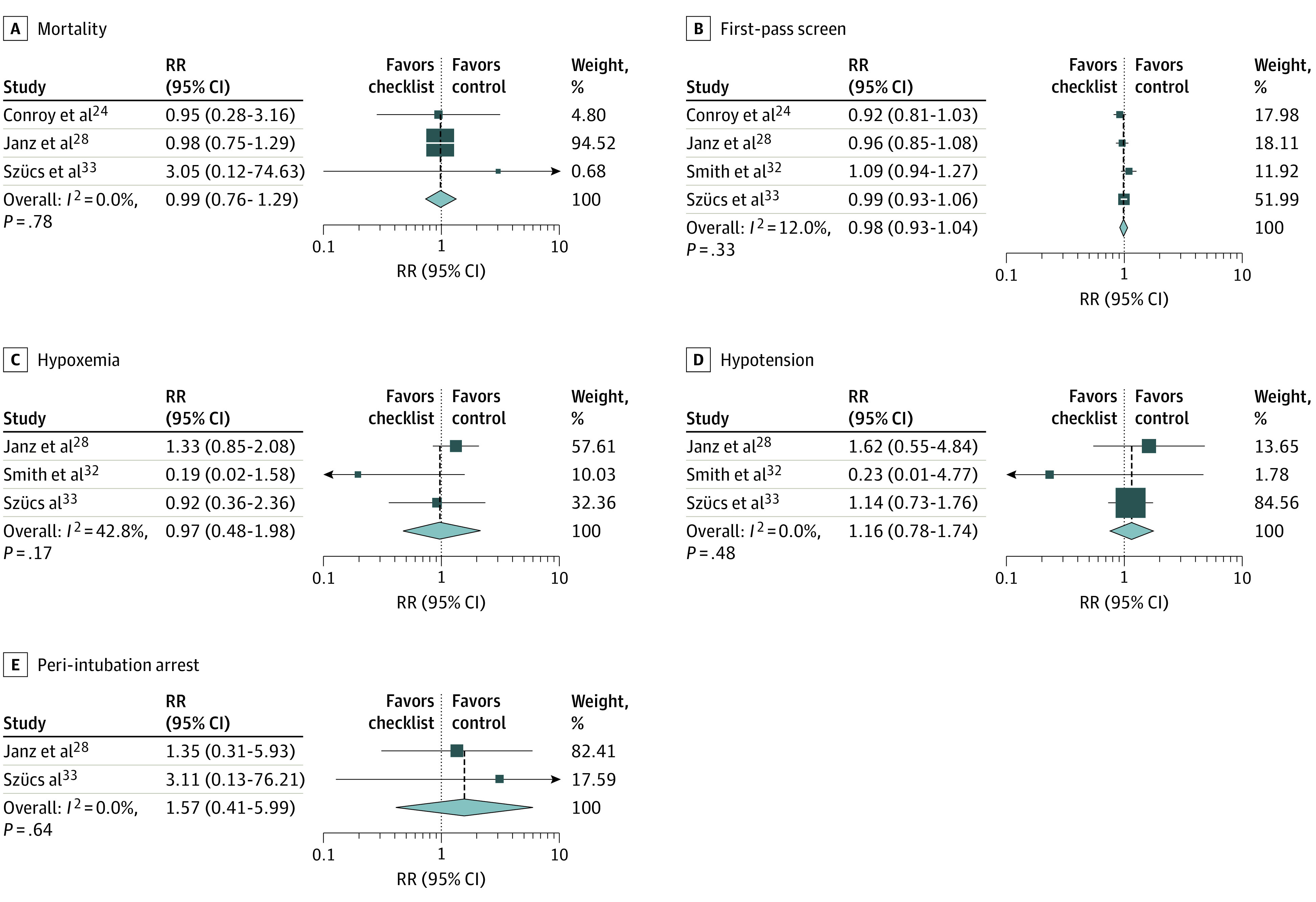
Low Risk of Bias Sensitivity Analysis Squares indicate relative risk (RR) estimates, with horizonal lines representing 95% CIs. Diamonds represent pooled estimates, with points indicating 95% CIs. Vertical dashed lines represent the relationship of the 95% CIs around each individual study result with the pooled mean. Weights are from random-effects analysis. A, E, and F, The study by Smith et al^32^ was not included in the analysis.

Figure 3 displays the results of the pooled analysis of the 4 studies at low risk of bias. In low-risk analyses, airway checklist use was not associated with improvement in any outcome. The nominal but nonstatistically significant suggestion of benefit seen in several primary analyses was absent or reversed in analyses of low risk of bias.

Subgroup analyses of ED vs ICU studies are presented in eFigure 2 in the Supplement, representing data from 7 ED studies^1,24,26,29,30,31,32^ and 3 ICU studies.^25,27,28^ No association with survival was found in either setting. For other adverse events, nominal estimates that suggested benefit were more marked in ED-based studies^1,24,26,29,30,31,32^ than ICU-based studies.^25,27,28^ In the ED studies,^1,24,26,29,30,31,32^ checklist use was associated with a decrease in hypoxia (RR, 0.61; 95% CI, 0.44-0.83) and esophageal intubation (RR, 0.07; 95% CI, 0.01-0.55). No individual outcomes were statistically significantly different between groups in the ICU studies.^25,27,28^

Three studies^27,29,30^ were performed in pediatric settings, and 8 studies^1,24,25,26,28,31,32,33^ contributed data from primarily adult settings.No differences in any adverse events were found in analyses limited to adult studies. In pediatric studies, checklist use was associated with decreased hypoxia (RR, 0.70; 95% CI, 0.57-0.86) but no other outcomes (eFigure 3 in the Supplement).

## Discussion

We identified 1 randomized clinical trial^28^ and 10 observational studies^1,24,25,26,27,29,30,31,32,33^ that compared clinical outcomes in ETI associated with and without an airway checklist. Summary estimates found no association between checklist use and mortality or most secondary outcomes, with the exception of decreased hypoxia. However, this association was not present in the sensitivity analysis of only studies with low risk of bias.^28,32,33^ Similarly, nominal but nonstatistically significant estimates that suggest benefit in several secondary outcomes were not apparent in sensitivity analyses of low risk of bias. Subgroup analyses suggested that checklist use may be more likely to be associated with decreased adverse events in pediatric settings and EDs compared with adult and ICU settings.

ETI is a high-risk procedure.^3,4,5,6,7,8^ Given that checklists have been associated with improved outcomes in other areas of health care,^8,9,10,11,12,13,35^ some have endorsed them for use with ETI.^6,14,15,16,36^ However, limited evidence supports such recommendations. Cabrini et al^37^ performed a systemic review of randomized clinical trials that evaluated any drug, technique, or device aimed at improving ETI. Similar to our review, the only randomized clinical trial that they identified that evaluated checklist use was the study by Janz et al,^28^ which found no benefit in any clinical outcomes. Hardy and Horner^38^ completed a “short-cut review” and concluded that checklists were likely beneficial, but further evidence was needed. That review, which was completed before several of the studies included in our review were published, included a conference abstract^39^ and 3 observational studies,^24,29,32^ 1^29^ of which had high risk of bias. Despite this lack of evidence, checklists for ETI are widely recommended.^6,14,15,16^

After pooling results from 11 different studies^1,24,25,26,27,28,29,30,31,32,33^ with more than 3000 patients, the only benefit statistically associated with checklist use in our systematic review was decreased hypoxic events. Because of the heterogeneity of hypoxia cutoffs in the included studies,^1,24,25,26,27,28,29,30,31,32,33^ hypoxia subgroups were defined based on the study definition of hypoxia. Results from studies^26,29,30,32,33^ using a cutoff of 90% or higher found an association with decreased hypoxia in patients intubated with use of a checklist, whereas this association was not observed in studies^25,27,28^ that examined more severe hypoxia (cutoffs of 60%-80%). More importantly, the finding of decreased hypoxia appeared to be primarily reported in studies with high risk of bias^25,26,27,28,29,30^ because the low risk of bias sensitivity analysis found no suggestion of decreased hypoxia. Most included studies^25,26,27,29,30,32,33^ used a before-and-after observational design. Such studies have high risk of bias for several reasons, including the Hawthorne effect, temporal improvement in care, and selection bias, and are prone to overestimation of effect sizes in favor of interventions.^40^ We found no suggestion of benefit for any outcomes when considering only studies with low risk of bias.^32,33^

Given the heterogeneity of patient populations and settings of the included studies,^1,24,25,26,27,28,29,30,31,32,33^ we performed subgroup analyses for adult and pediatric patients as well as ED and ICU settings. Subgroup analyses necessarily decrease the power to detect associations in each subgroup compared with the overall meta-analysis, and accordingly we found no statistically significant difference in most outcomes in subgroup analyses. Although only a few subgroup associations were apparent, some nominal suggestion of benefit was seen in several outcomes, with the estimates of effect generally more positive in pediatric and ED studies compared with adult and ICU studies. We propose that these differences are more likely related to the risk of bias in studies included in respective subgroups than to a true difference in effect size.

The current absence of evidence of benefit does not equate to a proven lack of benefit. It may be that checklists for ETI are associated with a decreased rate of rare, catastrophic events, such as peri-intubation cardiac arrest or cricothyrotomy. The number of patients required to define the effect of checklists on such rare events would be enormous. Although large, high-quality studies are needed to investigate checklist use further, randomized clinical trials of the size required to define precise effect estimates may not be feasible.

### Limitations

This study has limitations. No studies contained data for all the predefined outcomes, and no outcome was reported in more than 9 studies, with only 5 studies providing results for our primary outcome of mortality. This limitation contributed to wide 95% CIs around the effect estimates for many of our results. In some cases, the 95% CIs included the possibility of substantial benefit. Large sample sizes would be needed to have sufficient power to detect checklist benefit for rare events. One before-and-after observational study^34^ was not included in our analysis. This study^34^ used an intubating bundle protocol. We were unable to confirm whether a checklist was used during the intervention phase of the study; thus, it was excluded. The results of this excluded study^34^ were consistent with those of the meta-analysis, with a decrease in severe hypoxemia noted in the intervention period but no difference in mortality, esophageal intubation, or length-of-stay measures. Most contributing studies^1,24,25,26,27,29,30,31,32,33^ were observational and frequently included multiple cointerventions, further obscuring what associations could be attributed to checklist implementation. All the observational studies^1,24,25,26,27,29,30,31,32,33^ were case series or had before-and-after cohort designs, which are particularly prone to bias. Only 4^24,28,32,33^ of 11 included studies had low risk of bias. Estimates of effect in studies with low risk of bias were consistently less positive than in analyses that included all studies, suggesting that bias may have played a role in the results of higher-risk studies. Lastly, checklists may be more beneficial in settings that have low performance before implementation than in settings where the checklist items are already performed regularly at baseline. This information was not consistently available in the included studies. It is possible that academic centers, where studies are more likely to be performed, are already high performing, and checklist implementation would be more valuable in other settings.

## Conclusions

We found no association between survival and checklist use in patients undergoing ETI in this systematic review and meta-analysis. Checklist use was associated with a decrease in hypoxic events but no other secondary outcomes, although point estimates favored checklist use. Analyses of studies with low risk of bias found no association with decreased hypoxia, and point estimates did not suggest benefit. Additional high-quality studies in this area are needed, but current evidence does not support checklist use to improve clinical outcomes in patients undergoing ETI.

## Notes

Supplement. **eAppendix.** Search Strategy **eFigure 1.** Hypoxia Rates by Cutoff **eFigure 2.** Subgroup Analysis, ICU vs ED **eFigure 3.** Subgroup Analysis, Adult vs Pediatric Studies **eTable.** Commonly Used Elements of Airway Checklists
