# Error in Figure 2B

**DOI:** 10.1001/jamanetworkopen.2020.16899

**Published:** 2020-07-30

In the Original Investigation, “Association of Checklist Use in Endotracheal Intubation With Clinically Important Outcomes: A Systematic Review and Meta-analysis,”^1^ published on July 2, 2020, in Figure 2B, the labels above the graph were switched. This article has been corrected.

## References

[1] Turner JS, Bucca AW, Propst SL, . Association of checklist use in endotracheal intubation with clinically important outcomes: a systematic review and meta-analysis. JAMA Netw Open. 2020;3(7):e209278. doi:10.1001/jamanetworkopen.2020.9278 32614424 PMC7333022

