# Supplementary material for: Association of Checklist Use in Endotracheal Intubation With Clinically Important Outcomes: A Systematic Review and Meta-analysis
Source: JAMA Netw Open. 2020 Jul 2;3(7):e209278. doi: 10.1001/jamanetworkopen.2020.9278 (PMC7333022; doi:10.1001/jamanetworkopen.2020.9278)
Supplement: Supplement. — eAppendix. Search Strategy eFigure 1. Hypoxia Rates by Cutoff eFigure 2. Subgroup Analysis, ICU vs ED eFigure 3. Subgroup Analysis, Adult vs Pediatric Studies eTable. Commonly Used Elements of Airway Checklists [file jamanetwopen-e209278-s001.pdf]

Supplementary Online Content

Turner JS, Bucca AW, Propst SL, et al. Association of checklist use in endotracheal intubation with clinically important outcomes: a systematic review and meta-analysis. *JAMA Netw Open*. 2020;3(7):e209278. doi:10.1001/jamanetworkopen.2020.9278

eAppendix. Search Strategy

eFigure 1. Hypoxia Rates by Cutoff

eFigure 2. Subgroup Analysis, ICU vs. ED

eFigure 3. Subgroup Analysis, Adult vs. Pediatric Studies

eTable. Commonly Used Elements of Airway Checklists

This supplementary material has been provided by the authors to give readers additional information about their work.

© 2020 Turner JS et al. *JAMA Network Open*.

eAppendix – Search Strategy

#1
Search: (((("airway management"[MeSH Terms] OR ("airway"[All Fields] AND "management"[All Fields]) OR "airway management"[All Fields]) OR "Intubation, Intratracheal"[Mesh]) OR "Airway Management"[Mesh]) OR ("intubation"[MeSH Terms] OR "intubation"[All Fields])))
156,069 Results

#2
Search: (((("Task Performance and Analysis"[Mesh] OR ("checklist"[All Fields] OR "checklist"[All Fields])) OR parameter[All Fields]) OR "Checklist"[Mesh]) OR "Quality Improvement"[Mesh])
156,459 Results

#3
Search: #1 AND #2
2,129 Results

© 2020 Turner JS et al. *JAMA Network Open*.

eFigure 1 - Hypoxemia Rates by Cutoff

Cutoffs 90-93%

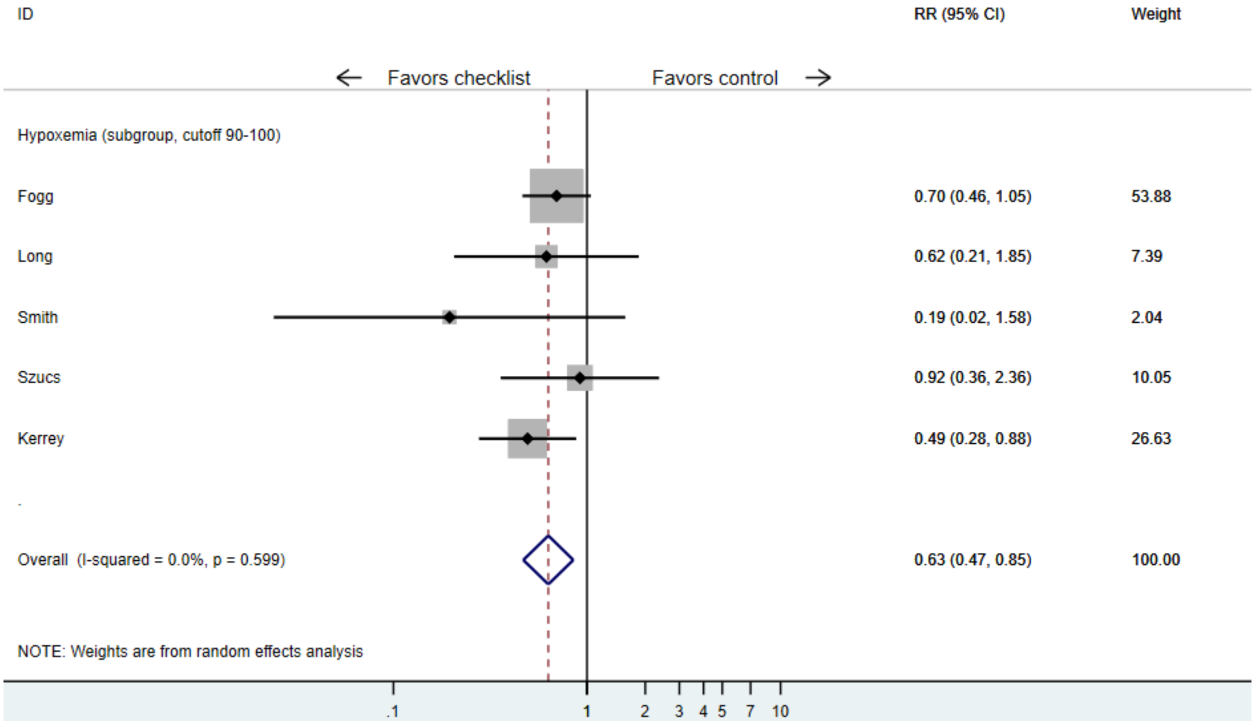

© 2020 Turner JS et al. *JAMA Network Open*.

Cutoffs 60-80%

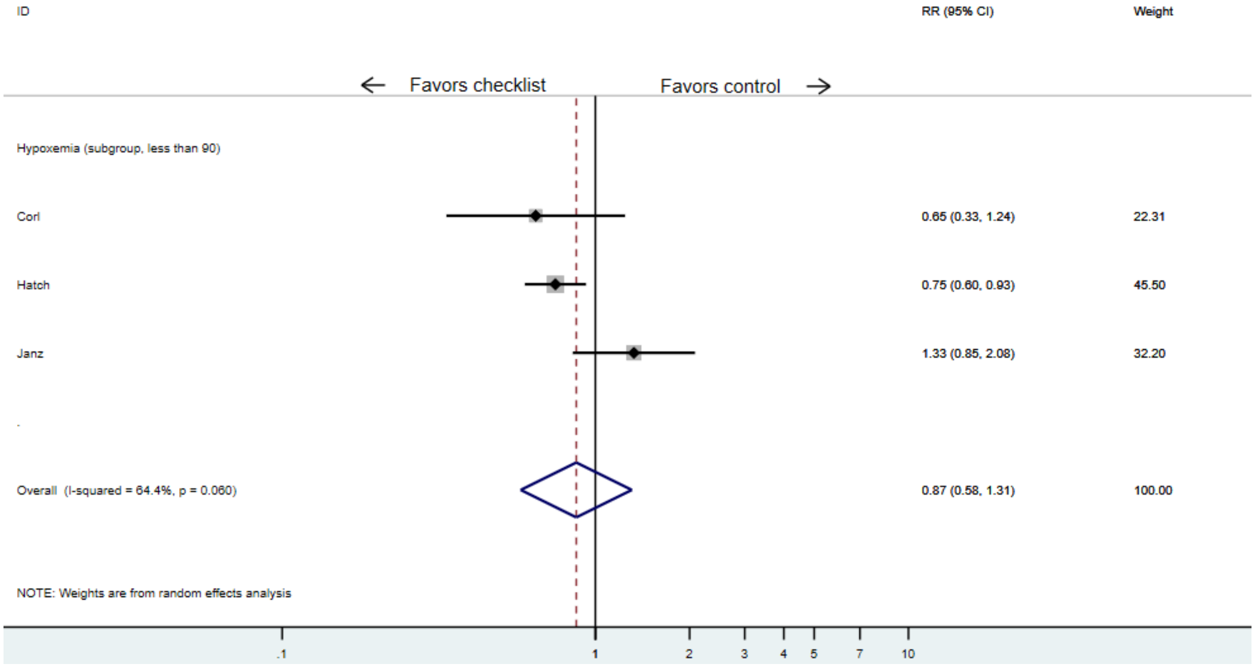

eFigure 2 – Subgroup Analysis, ICU vs. ED

MORTALITY

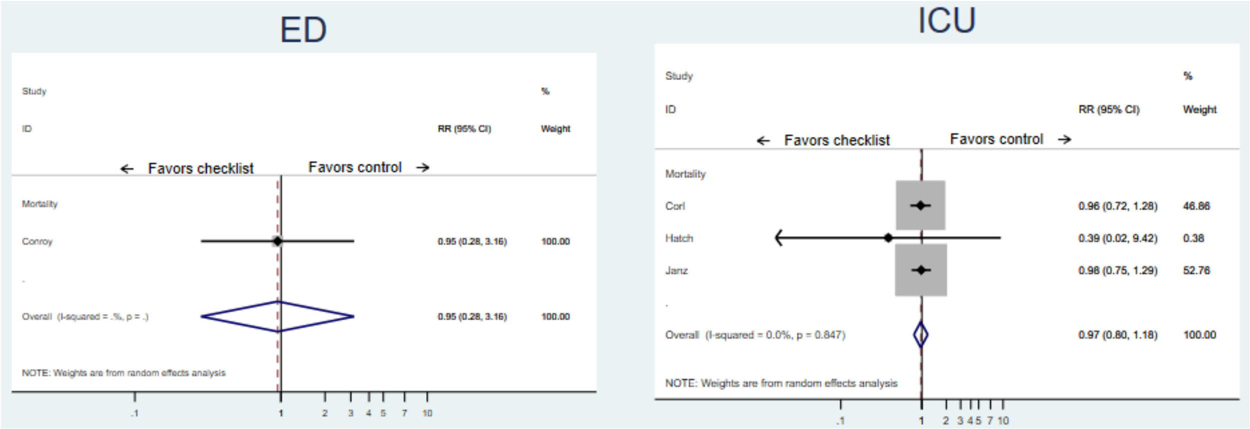

FIRST PASS SUCCESS

© 2020 Turner JS et al. *JAMA Network Open*.

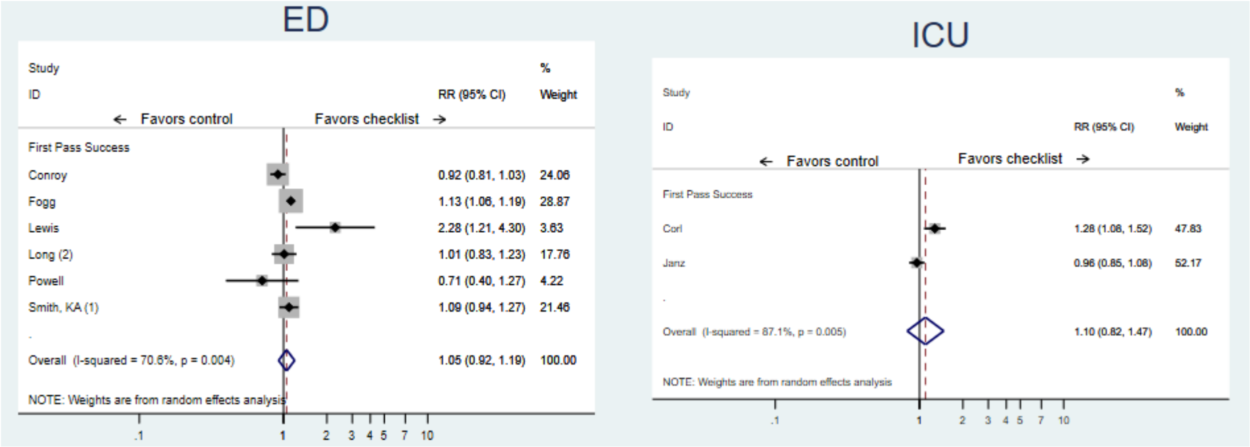

HYPOXEMIA

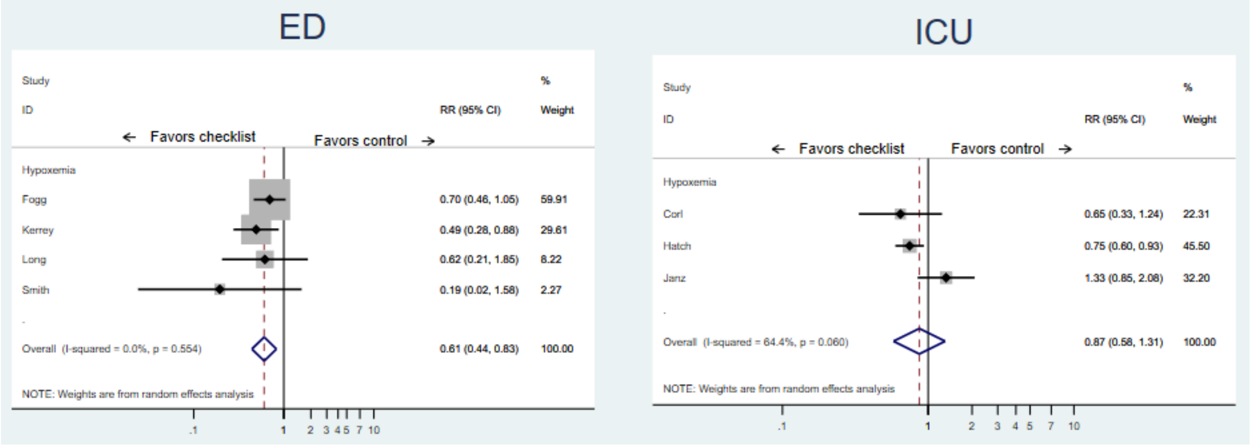

HYPOTENSION

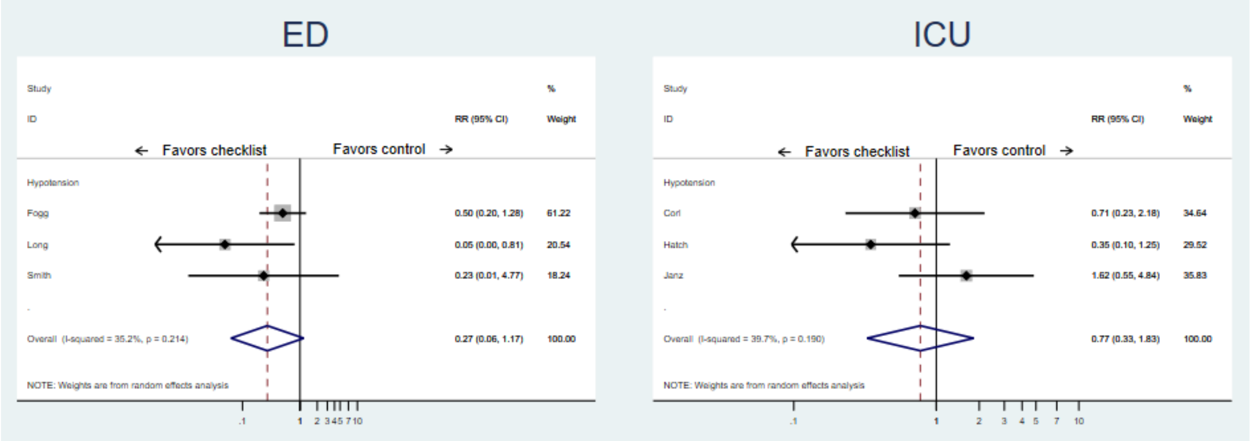

PERI-INTUBATION CARDIAC ARREST

© 2020 Turner JS et al. *JAMA Network Open*.

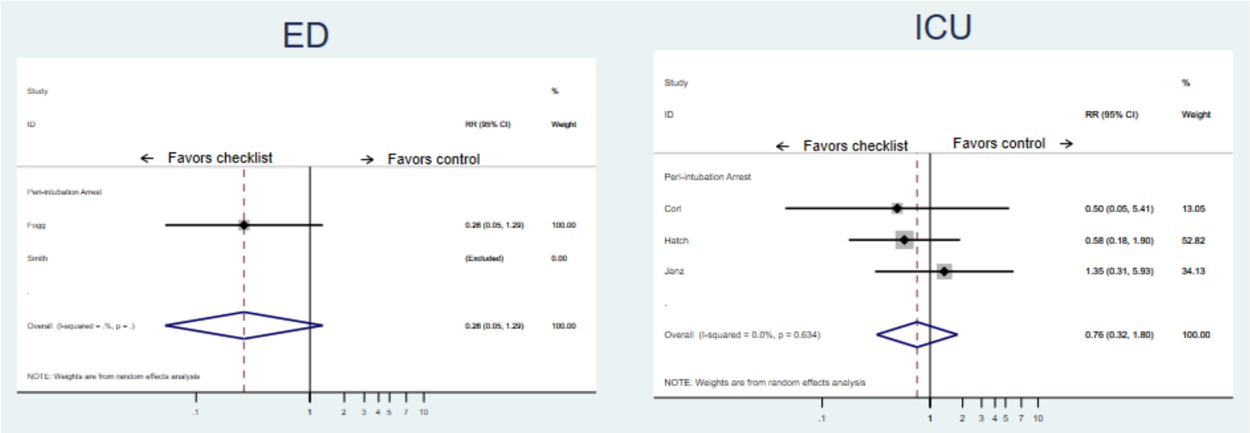

ESOPHAGEAL INTUBATION

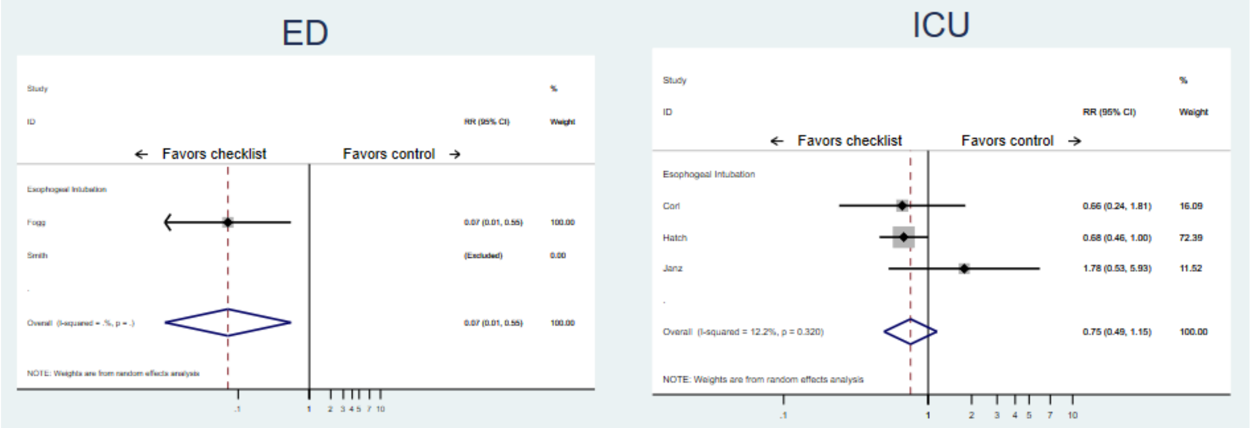

eFigure 3 – Subgroup Analysis, Adult vs. Pediatric Studies

MORTALITY

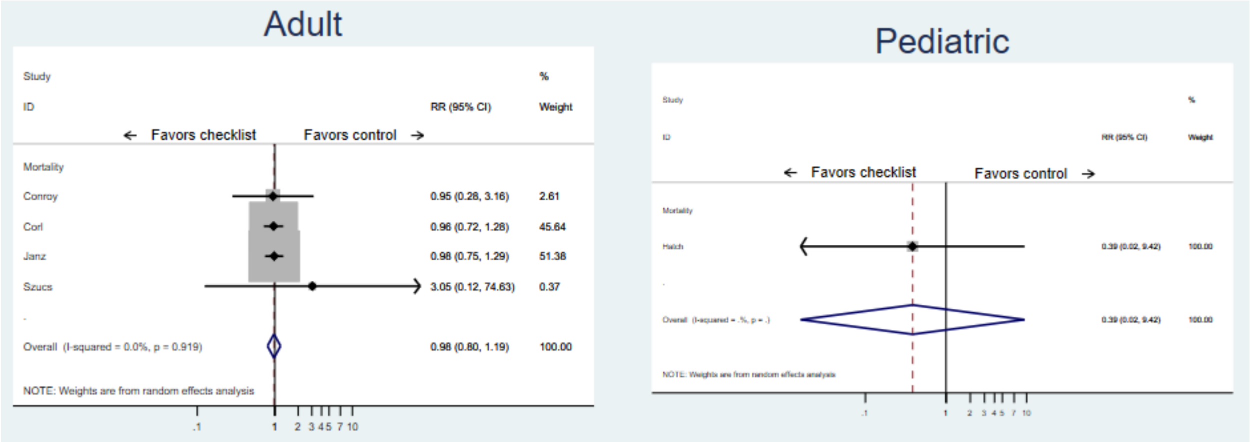

© 2020 Turner JS et al. *JAMA Network Open*.

FIRST PASS SUCCESS

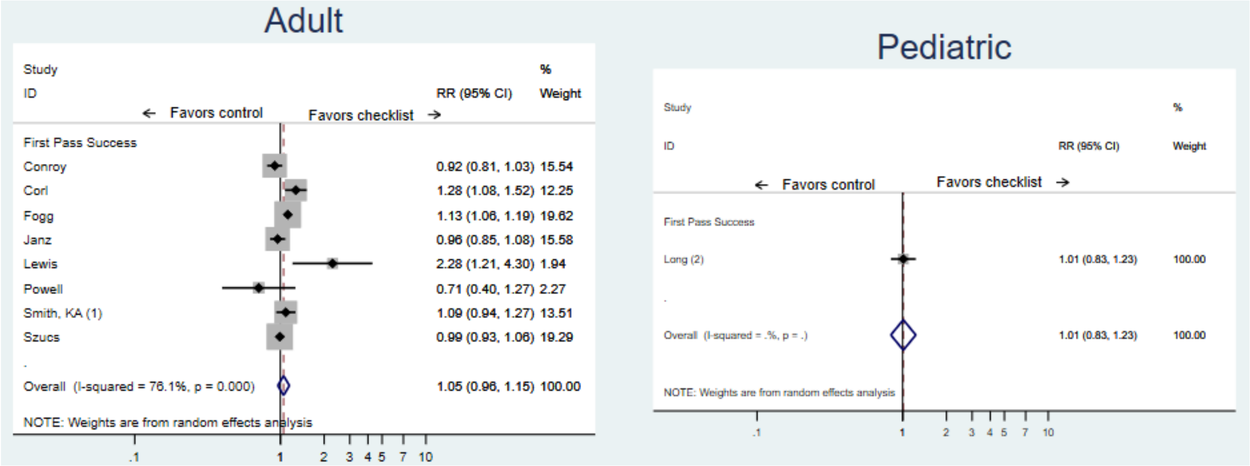

HYPOXEMIA

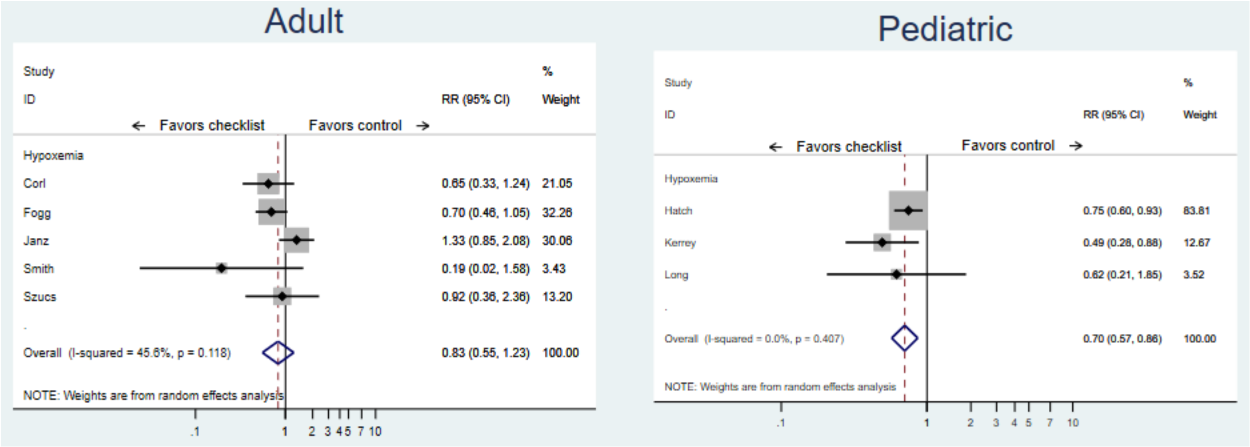

HYPOTENSION

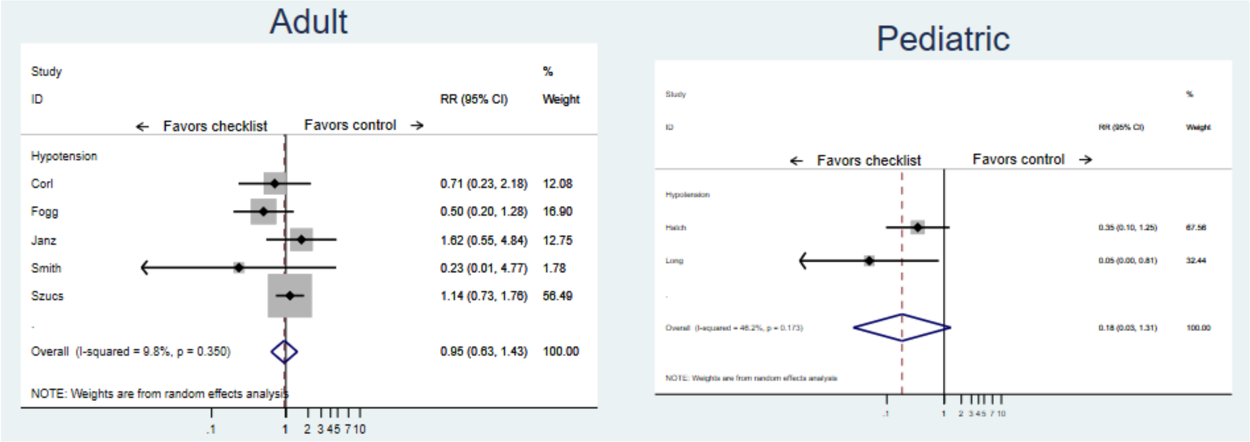

PERI-INTUBATION CARDIAC ARREST

© 2020 Turner JS et al. *JAMA Network Open*.

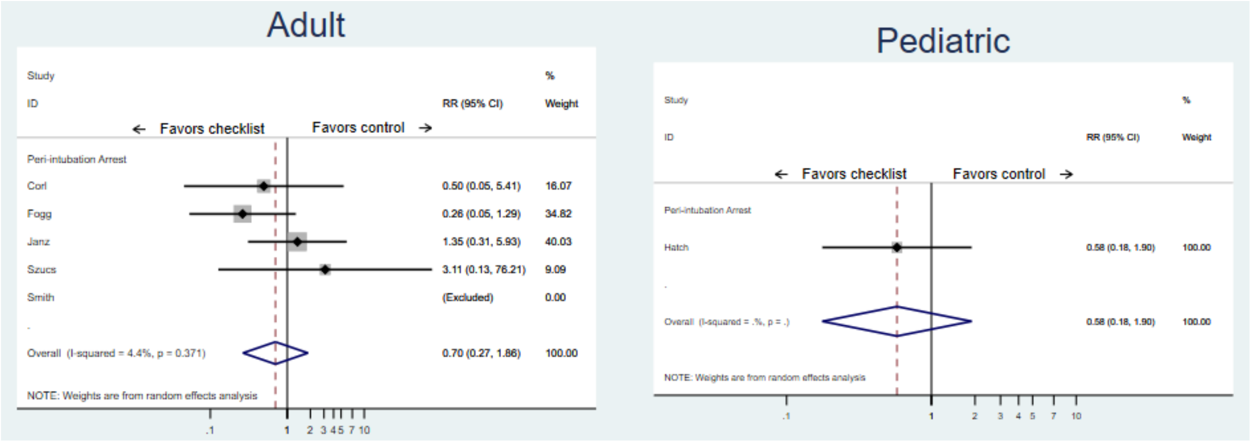

ESOPHAGEAL INTUBATION

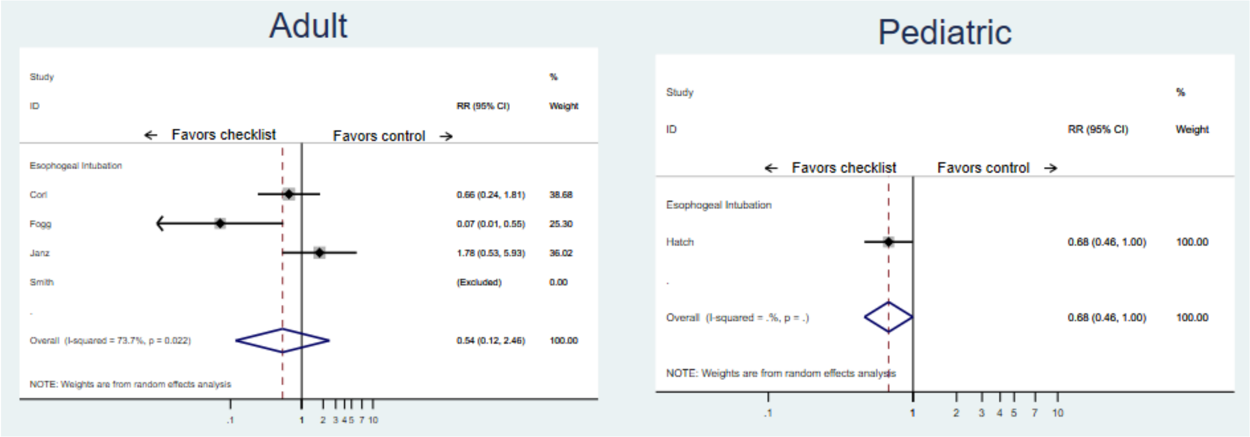

© 2020 Turner JS et al. *JAMA Network Open*.

eTable – Commonly Used Elements of Airway Checklists

Study	Preoxygenation	Medication Check	Capnography	Paralytics	Intubating Device check (video)	Fluid Bolus given or available	Intubator experience required	Suction check	Verbalize Plan
Conroy	X	X		X	X				
Corl	X	X	X	X		X			
Fogg	X				X		X		
Hatch		X			X				
Janz	X	X	X		X			X	X
Kerrey	X	X	X	X			X		X
Lewis*									
Long*									
Powell*									
Smith	X		X		X	X		X	X

*Details not provided in study

© 2020 Turner JS et al. *JAMA Network Open*.
